# Comparing International Guidelines for the Remission of Hypertension After Bariatric Surgery

**DOI:** 10.3390/clinpract15010011

**Published:** 2025-01-02

**Authors:** Carina Vieira Dias, Ana Lúcia Silva, Joana Dias, Paulo Cardoso, Rute Castanheira, Andreia Fernandes, Filipa Nunes, Tina Sanai, Mercedes Sanchez, João Maia-Teixeira, Ana Luísa De Sousa-Coelho

**Affiliations:** 1 Escola Superior de Saúde, Universidade do Algarve (ESSUAlg), 8005-139 Faro, Portugal; 2 Insight: Piaget Research Center for Ecological Human Development, Instituto Piaget, Av. João Paulo II, 1950-157 Lisboa, Portugal; 3 Health & Technology Research Center (H&TRC), Escola Superior de Tecnologia da Saúde de Lisboa (ESTeSL), Instituto Politécnico de Lisboa, 1990-096 Lisboa, Portugal; 4 Serviço de Nutrição, Hospital Cruz Vermelha (HCV), Rua Duarte Galvão, 1500-048 Lisboa, Portugal; 5 Faculdade de Medicina e Ciências Biomédicas (FMCB), Universidade do Algarve, 8005-139 Faro, Portugal; 6 Unidade Local de Saúde do Algarve (ULSALG), Unidade de Faro, Serviço de Cirurgia, Rua Leão Penedo, 8000-286 Faro, Portugal; 7 Algarve Biomedical Center Research Institute (ABC-Ri), Universidade do Algarve, 8005-139 Faro, Portugal

**Keywords:** hypertension, guidelines, remission, obesity, bariatric surgery, metabolic surgery

## Abstract

Background/Objectives: Obesity remains a global health concern and is associated with increased risk of type 2 diabetes, hypertension, and cardiovascular disease overall. Dissimilar hypertension guidelines are available for clinicians, namely those prepared by the American Heart Association (AHA) and the European Society of Cardiology (ESC), which may lead to distinctive appreciation of health outcomes of patients with obesity after bariatric and metabolic surgery, such as hypertension remission. The main goal of this study was to compare the effects of applying stricter (AHA) versus looser (ESC) blood pressure criteria on hypertension diagnosis pre-bariatric surgery and remission assessment one year post-op. Methods: A retrospective analysis of clinical data from patients who underwent surgical treatment for obesity at a single university hospital was performed. To evaluate the hypertension improvement or remission, two different types of blood pressure (BP) categorization were considered (based on AHA and ESC guidelines), in which each patient would fit according to their BP values pre- (m0) and 12 months postoperative (m12). Results: From a sample of 153 patients submitted for surgical treatment of obesity, more patients were considered with hypertension based on the AHA guideline (130 vs. 102; *p* < 0.001), while a higher rate of hypertension remission at 12 months after bariatric surgery was observed when following the ESC guideline (58.82 vs. 53.08%). Baseline patients’ clinical characteristics based on each hypertension outcome were mostly independent of the guideline used (*p* > 0.05), where only age and systolic blood pressure were relatively higher in “ESC groups”. Conclusions: We conclude that only minor differences exist between the two guidelines used. If evaluated based on ESC guidelines, it is expected that less patients are considered with hypertension, and the remission rate may be, at least numerically, higher.

## 1. Introduction

Hypertension is defined as the level of blood pressure (BP) at which the benefits of treatment (either with lifestyle interventions or drugs) unequivocally outweigh the risks of treatment, as documented by clinical trials [1]. With aging, populations may adopt more sedentary lifestyles which leads to an increase in body weight and hypertension prevalence [1]. Hypertension is identified as an obesity-related comorbidity, which means that it can be directly caused by overweight/obesity or that overweight/obesity may contribute to the presence of this condition. There is a complex relationship between obesity, diet quality, and hypertension. For instance, the consumption of energy-dense foods and nutritionally unbalanced products, both low in fiber and high in saturated fat, salt, and sugar, is associated with a higher incidence of general and abdominal obesity, while also elevating the risk of developing hypertension [2]. By contrast, dietary approaches to stop hypertension, the DASH diet (high potassium, low sodium) [3,4,5], or high-quality diets such as the Mediterranean diet [6], were found to significantly decrease BP. Among many food components presented in both diets, numerous plant-based antioxidant and anti-inflammatory ingredients showed their preventive and therapeutic effectiveness in cardiovascular diseases [7,8], such as polyphenols from different subclasses, i.e., resveratrol, epigallocatechin gallate, quercetin, or isorhamnetin, which can be found in various common edible sources [9,10,11]. Individuals who consume higher-quality diets are also less likely to develop metabolic syndrome [12]. Importantly, obesity-related comorbidities are expected to improve or go into remission when the patients achieve effective and sustainable weight loss [13], which can be achieved by a bariatric surgical procedure, also referred to as metabolic surgery [14,15]. Hypertension remission is considered when office BP values are below a determined threshold, associated with discontinuation of all antihypertensive treatment, while hypertension improvement is when there is a decrease in dosage and/or number of antihypertensive medications or when a decrease in systolic or diastolic BP levels is observed, even while maintaining the use of medication, but not achieving remission.

According to the American Heart Association (AHA), hypertension is defined as systolic blood pressure (SBP) ≥ 130 mmHg or diastolic blood pressure (DBP) ≥ 80 mmHg [16]. According to the guidelines of the European Society of Cardiology (ESC), hypertension is defined as an office SBP ≥ 140 mmHg and/or DBP ≥ 90 mmHg [1,17]. The most up-to-date version of the ESC guidelines, published in 2024, refers to the management of elevated blood pressure and hypertension instead of just “arterial hypertension” [18], reflecting that cardiovascular risk is on a continuous scale. This recent version recommends considering a straight categorization of hypertension to aid treatment decisions, while the previous version (2018) classified BP in grades 1–3 hypertension according to office BP, which will still be used in this study (Table 1). Although both associations established three hypertension stages (AHA) or grades (ESC), the defined threshold limits for hypertension diagnosis are different (Table 1), leading to some discussion regarding the lack of international harmonization [19].

Both the National Institute for Health and Care Excellence (NICE) [20] and the International Society of Hypertension (ISH) [21] present the same limits established by the ESC. By contrast, the Japanese Society of Hypertension (JSH) highlights the goal of an office BP of <130/80 mmHg, for adults with hypertension and other comorbidities [22], similarly to that established by the Taiwan Society of Cardiology (TSOC) and the Taiwan Hypertension Society (THS) [23]. Undeniably, the optimal BP values for each individual should not be based exclusively on their BP levels but considered along the assessment of their cardiovascular risk. Indeed, despite the values established for hypertension diagnosis, distinct BP targets were previously suggested by ESC for low–moderate-risk (<140/90 mmHg) or high-risk (<130/80 mmHg) patients [24].

This study aimed to compare the impact of using stricter (AHA) versus less strict (ESC) blood pressure guidelines in classifying hypertension among patients with obesity before bariatric surgery and in assessing hypertension remission one year post-surgery. The novelty of the presented study is the exploration of how different guidelines might lead to different outcomes, impacting the use of healthcare resources. These results could be relevant for harmonizing treatment protocols and improving patient care on an international scale.

## 2. Materials and Methods

### 2.1. Sample Selection and Data Collection

A retrospective cohort study was conducted in a subgroup of 153 patients (83.7% female (n = 128)) who undewent bariatric surgery (53.6% Roux-en-Y gastric bypass (RYGB, n = 82); 46.4% sleeve gastrectomy (SG, n = 71)) between 2015 and 2020 in Centro Hospitalar Universitário do Algarve (CHUA), Faro (Portugal), currently Unidade Local de Saúde do Algarve (ULSALG). The study was approved by the Ethics Committee and authorized by the Administration Board of CHUA. Information about patients was obtained from the clinical records. For the initial selection of the population of the study, patients without complete information about blood pressure values (diastolic and/or systolic) and medication in use, at baseline (month zero, m0) and/or in month twelve (m12) after bariatric surgery, were excluded. Patients submitted to conversion of SG to RYGB, the removal of an intragastric balloon, and any other type of surgical procedure not including RYGB or SG were also excluded from analyses.

Demographic data (age, sex), type of surgery, and height were exclusively collected at baseline (m0). Body weight (BW), medication, and systolic and diastolic BP values were both collected at m0 and twelve months after surgery (m12). For each patient, one BP measurement was performed by an experienced nurse, using a clinically validated and calibrated automated device, prior to each medical appointment, following the recommendations for “patient preparation” from the ESC 2024 guidelines [18]. Patients were organized by age group: <40, 40–49, 50–59 and ≥60 years old. Body mass index (BMI) was obtained based on the formula BW (in kg) divided by the square of height (kg/m^2^). Excess body weight (preoperative weight—ideal weight to produce a BMI of 25) was calculated before surgery (EBW). Total BW loss in percentage (TBWL, %) was calculated by subtracting the BW at m12 from the BW at m0, dividing by BW at m0, multiplied by 100. Change in BP (in mmHg) was calculated by subtracting the BP at m12 from the BP at m0. Medication was classified considering the use or not of antihypertensive medication and the number of antihypertensive medications used by each patient, at both m0 and m12.

### 2.2. Blood Pressure Categories

To evaluate the possible hypertension improvement or remission, two different types of BP categorization were considered, in which each patient would fit according to their BP values pre- (m0) and postoperatively (m12). According to the American Heart Association (AHA), the 3 categories for hypertension were defined as High Blood Pressure Stage 1 (SBP 130–139 or DBP 80–90 mmHg), High Blood Pressure Stage 2 (SBP ≥ 140 or DBP ≥ 90 mmHg) and Hypertensive Crisis (SBP > 180 mmHg and/or DBP > 120 mmHg) (Table 1). Regarding the European Society of Cardiology (ESC), the 3 categories were Grade 1 Hypertension (SBP 140–159 mmHg and/or DBP 90–99 mmHg), Grade 2 Hypertension (SBP 160–179 mmHg and/or DBP 100–109 mmHg) and Grade 3 Hypertension (SBP ≥ 180 mmHg and/or DBP ≥ 110 mmHg) (Table 1). Individuals with previous clinical diagnoses taking antihypertensive medication were also considered *with hypertension*, even if the measured BP values did not belong to a category considered to be classed as hypertension [25,26]. Although no out-of-office BP measurements were considered for the analysis, the clinical diagnosis was made based on repeated office BP measurements on more than one visit.

### 2.3. Hypertension Improvement

Hypertension improvement at m12 was defined when the patients either discontinued antihypertensive therapy or reduced their dosage but remained with BP values in a category considered hypertension, or when their BP values changed to a level considered less severe in the categories considered at m0.

### 2.4. Hypertension Remission

Regarding AHA guidelines, hypertension remission was considered if, in m0 of the evaluation, the BP values were in a category considered hypertension and/or patients were taking antihypertensive medication (i.e., classified as with hypertension according to AHA), but in m12 patients showed systolic BP < 130 mmHg and diastolic BP < 80 mmHg, as well as having stopped (or were not) taking antihypertensive medication. According to ESC guidelines, hypertension remission was considered if, in the baseline evaluation, patients were in a category considered hypertension and/or were taking antihypertensive medication but, in month twelve of the evaluation, improved their BP values to <140/90 mmHg and stopped (or were not) taking antihypertensive medication.

### 2.5. Statistical Analysis

Continuous variables were presented as means and standard deviation (SD), and as medians and interquartile range (IQR). If normally distributed, such variables were compared using independent-sample t-tests or one-way ANOVA. Mann–Whitney or Kruskal–Wallis tests were used to compare 2 or more groups of variables without normal distribution, respectively. To assess the distribution of the variables, the Shapiro–Wilk and Kolmogorov–Smirnov tests were used; non-normal distribution was considered if a variable failed to meet the criteria for normality in at least one of the indicated tests. Differences between patients’ characteristics at baseline (m0) and m12 were compared using the nonparametric Wilcoxon test for paired samples. When simple linear regression was performed, Pearson correlation was obtained. Categorical variables were described as frequencies and percentages and were compared by using a χ^2^ test. For the statistical analysis, IBM SSP Statistics version 28.0.1.0 (142) and GraphPad Prism 8 were used. A *p* value below 0.05 was considered statistically significant.

## 3. Results

### 3.1. Characterization of the Sample at Baseline (m0) and 12 Months After Bariatric Surgery

**Figure 1:**
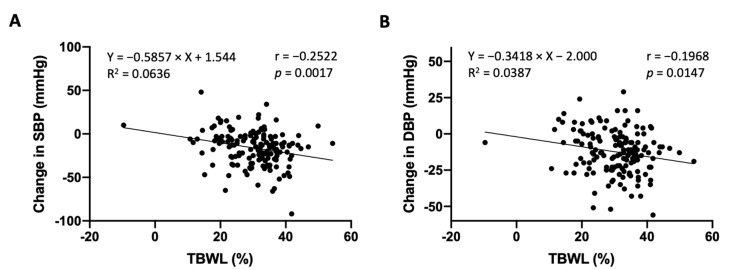
Higher body weight loss (TBWL) correlates with reductions in blood pressure: (**A**) TBWL (%) vs. change in SBP (mmHg). (**B**) TBWL (%) vs. change in DBP (mmHg). A simple linear regression was performed (equation and R squared are shown). Statistical analysis was performed using Pearson correlation (r). The respective *p* value is indicated in each graph. Abbreviations: SBP, systolic blood pressure; DBP, diastolic blood pressure.

The sample studied was composed of 153 patients who underwent bariatric surgery in a single center, with a mean age of 46.33 ± 10.57 years, followed over 1 year after the procedure. As anticipated, a statistically significant reduction in both BW and BMI was observed at m12 (Table 2), with an average of 30.32 ± 8.60% TBWL. At m0, mean SBP and DBP were, on average, 139.97 ± 19.38 mmHg (median 138.00 mmHg) and 80.48 ± 13.03 mmHg (median 81.00 mmHg), respectively. Both reduced to 123.76 ± 19.52 mmHg (median 121.00 mmHg) and 68.12 ± 11.51 mmHg (median 68.00 mmHg) at m12, respectively (Table 2). In this sample, the degree of TBWL modestly correlated with the drop in both SBP and DBP compared to baseline (Figure 1). From a total of 127 patients (83%) that were taking any kind of medication at baseline, more than half (58%, n = 74) were taking antihypertensive drugs (corresponding to 48.4% of the total sample). From these, most were taking two antihypertensive drugs (Table 2). At month twelve (m12), the number of patients using antihypertensive medication decreased to 13.7% (n = 21). Overall, there was a reduction in the number of patients taking any kind of medication at m12 (24.6%, n = 38), compared to m0 (Table 2).

### 3.2. Guideline-Dependent Hypertension Staging Based on Blood Pressure Levels

**Figure 2:**
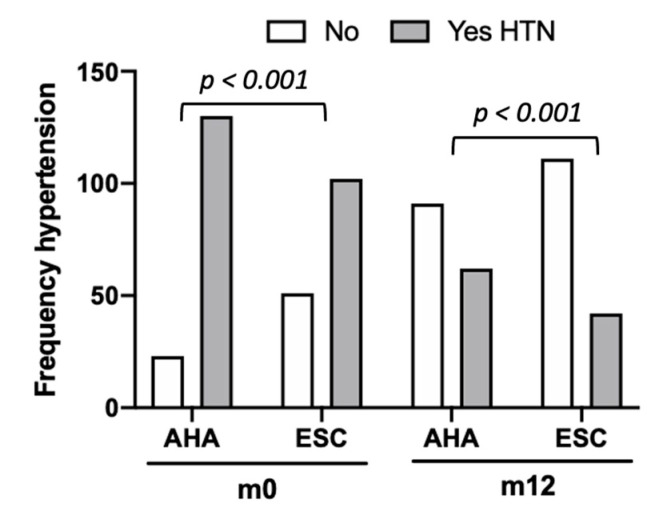
Frequency of patients classified with hypertension at baseline (m0) and 12 months after bariatric surgery (m12). Statistical analysis using χ^2^ test. The indicated *p* value corresponds to comparisons between guidelines for each timepoint (AHA vs. ESC). For each guideline, m0 vs. m12 *p* < 0.001. Abbreviations: AHA, American Heart Association; ESC Europe Society of Cardiology; HTN, hypertension.

Based on their BP levels and whether they were taking antihypertensive medication, patients were independently classified into AHA and ESC guideline categories (Table 1). Due to the higher limit of mmHg for a hypertension diagnosis, there were less individuals with hypertension at baseline according to ESC criteria (n = 102, 66.67%). By contrast, according to AHA classification, 85% (n = 130) were considered with hypertension (Figure 2, m0). According to ESC or AHA criteria, 40.5% (n = 62) or 27.5% (n = 42) were considered with hypertension at m12, respectively (Figure 2, m12).

For the AHA classification of hypertension, from the total sample, 28.8% (n = 44) had BP values of High Blood Pressure Stage 1, 44.4% (n = 68) were in the category High Blood Pressure Stage 2, and 3.9% (n = 6) were in the category Hypertensive Crisis (Table 3). At m12, a decrease in frequency was observed for all categories, being 17.0% (n = 26) in Stage 1, 15.7% (n = 24) in Stage 2, and 2.0% (n = 3) in the category Hypertensive Crisis (Table 3).

For ESC, also considering the total sample, for the categories considered hypertension, 48 (31.4%), 19 (12.4%), and 8 (5.2%) patients had, respectively, possible Hypertension Grades 1, 2, And 3 (Table 3). Based on such criteria, at m12, a decrease was observed in the categories considered hypertension, where nineteen (12.4%) patients had Grade 1, five (3.3%) had Grade 2, and three (2.0%) had BP values considered Grade 3 Hypertension (Table 3).

### 3.3. Remission of Hypertension Based on AHA or ESC Guidelines

**Figure 3:**
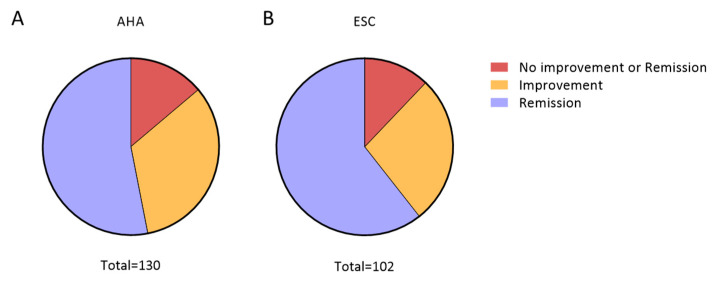
Distribution of hypertension outcomes regarding the two different guidelines: (**A**) Distribution based on AHA guideline. From a total of 130 patients, n = 69 (53.08%) showed remission; n = 43 (33.08%) improved; and n = 18 (13.85%) did not improve or showed remission). (**B**) Distribution based on ESC guideline. From a total of 102 patients, n = 60 (58.82%) showed remission; n = 30 (29.41%) improved; and n = 12 (11.76%) did not improve or showed remission). Comparing guidelines, *p* < 0.001; statistical analysis using χ^2^ test. Abbreviations: AHA, American Heart Association, ESC, Europe Society of Cardiology.

Considering the absolute numbers of patients with hypertension for each guideline, the improvement or remission of hypertension was evaluated at m12. According to AHA guidelines, 18 (13.85%) patients did not achieve hypertension improvement or remission at m12. Patients that improved (but did not achieve remission) corresponded to 33.08% (n = 43), and the remission rate was 53.08% (n = 69). According to ESC guidelines, 12 (11.76%) patients did not improve or show remission. The improvement rate was 29.41% (n = 30) and the remission rate was 58.82% (n = 60) (*p* < 0.001) (Figure 3).

**Figure 4:**
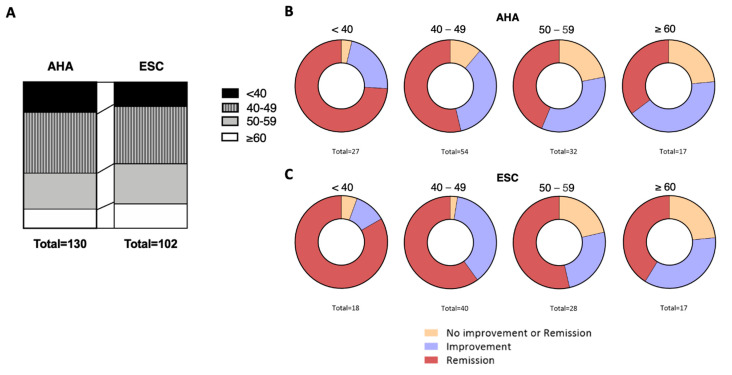
(**A**). Age distribution in decades for “AHA” and “ESC” patients: (**B**,**C**) Comparison between age groups regarding to the hypertension outcome. (**B**) AHA (*p* = 0.013); (**C**) ESC (*p* < 0.001); statistical analysis using χ^2^ test. Abbreviations: AHA, American Heart Association; ESC, European Society of Cardiology.

As for the patient characteristics, baseline variables of weight, BMI, EBW, and DBP were not statistically significant in terms of potentially influencing patient outcomes, independently of the guideline considered (Table 4). By contrast, the patients’ age and SBP at m0 were statistically different between the established outcomes, for both guidelines (Table 4). Indeed, patients that did not improve were older than those who showed hypertension remission. While the mean age of the “ESC patients” was numerically higher than “AHA patients” (49 vs. 47 years old, respectively, *p* = 0.1243), mostly because of a lower proportion of patients below 40 years of age and an increased proportion of those over 60 years of age (Figure 4A), there were no statistical differences between guidelines in any of the outcome groups (Table 4).

The distributions of the hypertension outcome by age group were statistically different for both guidelines (AHA and ESC, *p* = 0.013 and *p* < 0.001, respectively), evidenced by a decrease in the rate of remission when increasing age (Figure 4B,C).

## 4. Discussion

With the increasing prevalence of obesity worldwide and associated comorbidities, there is a growing interest in research to understand the impact of obesity treatment on its comorbidities. There is also an increasing interest from researchers to understand whether age and/or which age-dependent factors may interfere with the remission of obesity-associated comorbidities, namely hypertension. While age is non-modifiable, excess body weight is a modifiable risk factor [27,28]. Numerous studies have witnessed the positive effects of bariatric surgery on metabolic risk factors and multiple comorbidities. A higher remission rate of hypertension or reduction in the use of antihypertensives is reached in patients undergoing BS when compared with patients submitted to medical or lifestyle interventions [29,30]. While many studies have used the ESC guidelines for establishing hypertension diagnosis and remission [30,31,32], for BP goals and targets, there is some lack of consensus. For instance, in 2018, the American Academy of Family Physicians (AAFP) and the American College of Physicians (ACP) issued different recommendations [33], which were looser (<150 mm Hg for individuals > 60 years old) when compared to those by both the AHA and ESC. To identify the concordance of AHA and ESC classifications of hypertension, the present study aimed to compare the recent updated guidelines on the resultant improvement and remission of hypertension in individuals after surgical treatment of obesity. Indeed, while weight loss is consistently emphasized as an important non-pharmacological approach for blood pressure control across guidelines [34], the rationale was to perform a study comparing the outcomes in terms of hypertension improvements after bariatric and metabolic surgery, considering two main international guidelines.

Being categorized at baseline (before bariatric surgery) as *without hypertension* and *with hypertension*, according to the criteria of each guideline, the improvement and remission rates were calculated. The lower thresholds of AHA included more patients in the category *with hypertension*. By contrast, based on ESC guidelines, some patients (n = 28) were not classified *with hypertension*, being categorized as *high normal* BP values and not considered for the following analyses. The two definitions also led to different percentages of hypertension remission, namely 53.08% and 58.82%, for AHA and ESC, respectively. This is because by the AHA guidelines, hypertension remission is when SBP values are lower than 130 mmHg and the DBP values are lower than 80 mmHg, which shows a bigger restriction in the BP values compared to the ESC guideline (SBP < 140 mmHg and DBP < 90 mmHg). The categorization performed led to statistically significant differences in certain baseline variables between hypertension outcome groups, independently of the guideline used. Despite no statistically significant differences being identified when comparing guidelines, age and SBP, when assessed according to the AHA guidelines, the values showed higher statistical significance (i.e., lower *p* values). This might be important to highlight when reporting the success of metabolic surgery in terms of comorbidities remission, at the international level, as a different characterization of the patients may be obtained. While no major differences were identified in our comparative analysis, the comparison of recommendations prompts a reconsideration of harmonizing thresholds, particularly for specific populations such as patients living with obesity and elderly people. Nevertheless, it should also draw our focus toward individualized care, considering the unique characteristics and needs of each patient following bariatric and metabolic surgery, to achieve the best possible outcomes. As this manuscript was being prepared, new recommendations from the ESC were published. In 2018, the ESC recommended classifying blood pressure as optimal, normal, high normal, or grades 1–3 hypertension [1]. However, the 2024 ESC guidelines introduce a simplified three-category system: non-elevated BP; elevated BP; and hypertension [18]. The key modifications included consolidating the previous “normal” and “high-normal” categories into a single “elevated” category and eliminating the detailed grading system for hypertension stages. The present study maintained the 2018 ESC hypertension categories for two main reasons: (1) to use established cut-off values comparable to AHA guidelines and (2) to facilitate the concept of blood pressure improvements for comparison. Importantly, using either the new or old ESC guidelines does not affect the results obtained, as both define hypertension as SBP ≥ 140 mmHg or DBP ≥ 90 mmHg.

The mechanisms of obesity and obesity-related hypertension are complex and sometimes mutually dependent. The primary factors, in addition to genetic and environmental factors, are alterations to the sympathetic nervous system, renal and adrenal function, adipokine secretion, and insulin resistance [35]. These mechanisms combined, as well as a lower capacity to lose EBW, could lead to a diminished remission rate in older people. Although this study briefly assessed the effect of age, we did not focus on other comorbidities associated with obesity, nor did we analyze data regarding the patients’ exercise capability, smoking habits, salt consumption, or overall diet habits before and after surgery. The main limitations are that this is a study with a convenience patient selection, which led to a relatively small number of patients (mainly female), and the fact that it was an opportunistic screening, performed using office BP measurements to detect possible hypertension, which can hamper the interpretation of the results. Because of the small sample size, the statistical power of the study is reduced, making it more challenging to detect meaningful patterns or significant differences. The unequal sex distribution may also not accurately represent the population of interest, potentially skewing the results and limiting the generalizability. However, many other studies have encountered that the population of candidates for bariatric and metabolic surgery is predominately female, i.e., this sex disparity is documented in the previous literature [36], despite similar outcomes in terms of weight loss, remission of comorbidities, and incidence of surgical complications between sexes [37]. The main strength of this study is the fact that the main international guidelines were considered to assess the improvement and remission rates of the hypertensive patients. To the best of our knowledge, this is the first study that uses two guidelines to assess and compare hypertension remission after surgical treatment of obesity.

## 5. Conclusions

Patients with obesity and hypertension benefit from bariatric procedures in terms of reducing body weight and BP values. More than half of the patients achieved hypertension remission, independently of the guideline used, although the ESC-based remission rate was higher. We conclude that only minimal differences are observed between the two guidelines used. However, while globally reporting the impact of weight loss strategies on hypertension remission is valuable, establishing a personalized approach tailored to each patient’s individual BP goals is even more crucial.

## Figures and Tables

**Table 1:** AHA and ESC blood pressure categories.

	American Heart Association				European Society of Cardiology		
**Category**	Systolic Blood Pressure		Diastolic Blood Pressure	**Category**	Systolic Blood Pressure		Diastolic Blood Pressure
Normal	<120	and	<80	Non-elevated ^1^	<120	and	<70
Elevated	120–129	and	<80	Elevated ^1^	120–139	or	70–89
**Stage 1 Hypertension**	130–139	or	80–89				
**Stage 2 Hypertension**	≥140	or	≥90	**Grade 1 Hypertension ^2^**	140–159	and/or	90–99
				**Grade 2 Hypertension ^2^**	160–179	and/or	100–109
**Hypertension Crisis**	≥180	and/or	≥120	**Grade 3 Hypertension ^2^**	≥180	and/or	≥110

Abbreviations: American Heart Association, AHA; European Society of Cardiology, ESC. ^1^ According to the ESC 2024 guidelines. ^2^ According to the ESC 2018 guidelines. Values are in mmHg.

**Table 2:** Sample characterization at baseline (m0) and one year (m12) after obesity surgery.

Variable	Baseline (m0)	One Year After BS (m12)	*p* Value
**Body weight (kg)**, mean (SD) median (IQR)	111.83 (18.51) 108.8 (22.6)	77.42 (13.14) 75.70 (18.45)	*p* < 0.0001
**Body mass index (kg/m^2^)**, mean (SD) median (IQR)	41.56 (5.06) 41.29 (5.64)	28.82 (4.03) 28.10 (5.75)	*p* < 0.0001
**Systolic BP (mmHg)**, mean (SD) median (IQR)	139.97 (19.38) 138.00 (25.50)	123.76 (19.52) 121.00 (24.50)	*p* < 0.0001
**Diastolic BP (mmHg)**, mean (SD) median (IQR)	80.48 (13.03) 81.00 (15.00)	68.12 (11.51) 68.00 (14.00)	*p* < 0.0001
**Medication**, n (%), Yes	127 (83.01)	38 (24.6)	*p* < 0.001
**Antihypertensive medication**, n (%), Yes	74 (48.4)	21 (13.7)	*p* < 0.001
**Number of antihypertensive medications, n (%)**			*p* < 0.001
No antihypertensive medication	79 (51.6)	132 (86.3)	
1 medicine	20 (13.1)	6 (3.9)	
2 medicines	39 (25.5)	8 (5.2)	
3 medicines	9 (5.9)	1 (0.7)	
4 medicines	5 (3.3)	3 (2.0)	
Missing information about the number	1 (0.7)	3 (2.0)	

Statistical analysis using χ^2^ test for categorical variables. Wilcoxon test was used to compare continuous variables. Abbreviations: BS, bariatric surgery; RYGBP, Roux-Y Gastric Bypass; SG, sleeve gastrectomy; AHA: American Heart Association; ESC, European Society of Cardiology; BP, blood pressure. SD, standard deviation; IQR, interquartile range.

**Table 3:** AHA and ESC hypertension categories.

	American Heart Association				European Society of Cardiology		
**Category**	m0, n (%)		m12, n (%)	**Category ^1^**	m0, n (%)		m12, n (%)
**Stage 1**	44 (28.8)		26 (17.0)	**Grade 1**	48 (31.4)		19 (12.4)
**Stage 2**	68 (44.4)		24 (15.7)	**Grade 2**	19 (12.4)		5 (3.3)
**Crisis**	6 (3.9)		3 (2.0)	**Grade 3**	8 (5.2)		3 (2.0)

For each guideline, m0 vs. m12 *p* < 0.001; statistical analysis using χ^2^ test. ^1^ According to the ESC 2018 guidelines. Abbreviations: American Heart Association, AHA; European Society of Cardiology, ESC.

**Table 4:** Baseline (m0) differences in frequencies, means, and standard deviations (SDs) of sex, age, weight, body mass index, excess body weight, and systolic and diastolic blood pressure between each blood pressure criteria at m12 (total, no improvement/remission, improvement, and remission groups).

		n (%)	Female, n (%)	Age (y), Mean (SD)	Weight (kg), Mean (SD)	BMI (kg/m^2^), Mean (SD)	EBW (kg), Mean (SD)	SBP (mmHg), Mean (SD)	DBP (mmHg), Mean (SD)
**Total**	AHA	130 (100)	107 (82.3)	47.4 (10.3)	112.7 (19.0)	41.9 (5.1)	45.5 (15.2)	143.8 (18.3)	82.5 (12.8)
	ESC	102 (100)	81 (79.4)	49.4 (9.6)	112.5 (19.5)	41.9 (5.4)	45.5 (16.0)	146.9 (19.3)	83.9 (13.2)
	***p *** **value**	NA	NA	0.124	0.861 ^MW^	0.883 ^MW^	0.862 ^MW^	0.127 ^MW^	0.398 ^MW^
**No improvement or remission**	AHA	18 (13.8)	16 (88.9)	53.6 (10.1)	108.6 (15.8)	40.5 (5.0)	41.5 (13.8)	140.1 (14.7)	77.8 (11.5)
	ESC	12 (11.8)	10 (83.3)	57.3 (10.1)	109.0 (17.7)	40.5 (5.2)	41.7 (14.8)	145.2 (15.2)	78.8 (12.3)
	***p *** **value**	NA	NA	0.342	0.941	0.986	0.973	0.365	0.837
**Improvement**	AHA	43 (33.1)	37 (86.1)	49.7 (8.9)	113.0 (18.5)	42.8 (5.6)	47.0 (15.7)	154.4 (18.5)	85.3 (12.4)
	ESC	30 (29.4)	25 (83.3)	50.5 (8.6)	115.0 (19.9)	43.7 (5.9)	49.3 (16.7)	156.2 (20.6)	84.6 (13.5)
	***p *** **value**	NA	NA	0.696	0.678 ^MW^	0.591 ^MW^	0.551	0.740 ^MW^	0.736 ^MW^
**Remission**	AHA	69 (53.1)	54 (78.3)	44.4 (10.3)	113.6 (20.1)	41.7 (4.8)	45.7 (15.3)	138.2 (16.2)	82.0 (13.1)
	ESC	60 (58.2)	46 (76.7)	47.4 (9.2)	112.0 (19.8)	41.3 (5.1)	44.4 (15.7)	142.5 (18.1)	84.7 (13.2)
	***p *** **value**	NA	NA	0.086	0.619 ^MW^	0.645 ^MW^	0.589 ^MW^	0.100 ^MW^	0.252
*p* value (3 groups)	AHA	NA	0.393	**0.0005**	0.600 ^KW^	0.457 ^KW^	0.461 ^KW^	**<0.001** ^KW^	0.136 ^KW^
	ESC	NA	0.184	**0.003**	0.494 ^KW^	0.162 ^KW^	0.208 ^KW^	**0.011** ^KW^	0.356

Abbreviations: AHA, American Heart Association; ESC, European Society of Cardiology; BMI, body mass index; EBW, excess body weight; SBP, systolic blood pressure; DBP, diastolic blood pressure. NA, not assessed. Statistical analyses were performed using χ^2^ test for all categorical variables. For continuous variables, t test or Mann–Whitney test (indicated at *p* value by superscript ^MW^) was performed when data were normally or not normally distributed, respectively, when comparing AHA vs. ESC. ANOVA or Kruskal–Wallis (superscript ^KW^) tests performed when comparing the 3 groups (no improvement or remission; improvement; remission), as indicated in the line *p* value (3 groups), depending on data being normally or not normally distributed, respectively. *p* values below 0.05 are indicated in bold. Background grey highlights specific rows, namely those for total values and when comparing the three groups (no improvement or remission, improvement, and remission) for each guideline, and columns, namely the total n and respective percentage.

## Data Availability

The data supporting the findings of this study are available on reasonable request to the corresponding author.
